# Community pharmacists’ engagement with Raqeeb platform: perceptions and implementation challenges toward controlled substance monitoring in Saudi Arabia

**DOI:** 10.3389/fphar.2025.1644493

**Published:** 2025-09-25

**Authors:** Sultan M. Alshahrani

**Affiliations:** Department of Clinical Pharmacy, College of Pharmacy, King Khalid University, Abha, Saudi Arabia

**Keywords:** Raqeeb platform, community pharmacists, NDPS monitoring, gabapentinoids, Saudi Arabia

## Abstract

**Introduction:**

The misuse of narcotic drugs and psychotropic substances (NDPS), especially gabapentinoids, poses a considerable public health concern in Saudi Arabia, leading to the establishment of the Raqeeb platform, a nationwide electronic platform for the real-time monitoring of NDPS prescriptions. This study aimed to assess community pharmacists’ perceptions, engagement, and implementation challenges with the Raqeeb platform in order to inform future improvements.

**Methods:**

A cross-sectional descriptive study was performed from March to May 2025, targeting licensed community pharmacists in Saudi Arabia. A standardized, self-administered questionnaire, validated by experts and piloted with 15 pharmacists, evaluated knowledge, attitudes, and operational challenges using a five-point Likert scale. Convenience sampling through professional networks and social media produced 270 responses. Data were analyzed with IBM SPSS Statistics version 26, employing descriptive statistics and Cronbach’s alpha to assess reliability.

**Results:**

Participants (58.1% male, 62.2% PharmD, 78.1% with less than 5 years of experience) demonstrated strong awareness of Raqeeb’s objectives and its role in enhancing safety. Most respondents supported mandatory training and nationwide expansion of the platform. However, challenges were reported, including limited integration with pharmacy systems, complexity of the interface, and unavailability of certain controlled drugs. Cronbach’s alpha values (0.73–0.87) confirmed the instrument’s reliability.

**Conclusion:**

Community pharmacists recognize the public health value of Raqeeb; however, its best use has been limited by issues with usability, integration, and training. Addressing these barriers through system integration, structured training, and improved medication availability is essential to strengthening NDPS monitoring and advancing Vision 2030 digital health goals.

## 1 Introduction

The misuse of narcotic drugs and psychotropic substances (NDPS), especially gabapentinoids such as pregabalin and gabapentin, has emerged as a major public health concern globally. While these drugs are recommended for neuropathic pain and epilepsy, rising off-label usage and diversion for recreational purposes have been reported in numerous countries (Evoy et al., 2021; Hägg et al., 2020; Chiappini and Schifano, 2016). Pregabalin, in particular, has shown substantial misuse potential due to its rapid onset of action and sedative–euphoric characteristics, especially when co-administered with opioids and benzodiazepines (Pharmacopoeia, 2013; Kurdi, 2021).

In response, several nations have introduced regulatory measures to restrict access to gabapentinoids. The United Kingdom reclassified pregabalin and gabapentin as Class C controlled substances in 2019 following a notable increase in fatalities associated with their use (UK Home Office, 2025). Similarly, France, Germany, and Sweden have tightened regulatory oversight through national databases or scheduling under controlled drug frameworks (Driot et al., 2019; Finklea et al., 2025). In the United States, over 22 states now apply restrictions on dispensing gabapentinoids, which must be reported to Prescription Drug Monitoring Programs (PDMPs) (Karavolis et al., 2022).

In Saudi Arabia, escalating misuse, particularly among young adults and individuals with psychiatric comorbidities, led to the reclassification of pregabalin and gabapentin as controlled drugs in 2017 (Alkhalaf et al., 2021; Althunian et al., 2022). To enhance prescription surveillance, the Saudi Food and Drug Authority (SFDA) implemented the Raqeeb platform, an integrated national electronic system that enables real-time monitoring of NDPS prescribing and dispensing across all healthcare sectors (Aldossari et al., 2025). Raqeeb is part of Saudi Arabia’s broader digital health transformation under Vision 2030, where platforms such as Seha support the integration of electronic health services and strengthen accountability in the handling of controlled substances (Al-Kahtani et al., 2022).

This initiative aligns with Saudi Arabia’s Vision 2030 strategy, which emphasizes the digital transformation of healthcare to improve transparency, safety, and regulatory compliance. Comparable systems, such as SafeScript in Australia and the Electronic Prescription Service (EPS) in the UK, have demonstrated improvements in medication safety and prescriber accountability (Goundrey-Smith, 2018; Sheikh et al., 2022). However, successful implementation depends heavily on the engagement and capacity of frontline providers, especially community pharmacists.

Community pharmacists in Saudi Arabia serve as gatekeepers in NDPS dispensing and are increasingly expected to integrate surveillance platforms like Raqeeb into their workflow. In a recent study, Alshahrani et al. (Alshahrani et al., 2021) highlighted that Saudi pharmacists play a growing role in pharmaceutical care, including medication review, counseling, and adherence to regulatory requirements. Their involvement is particularly critical given the widespread accessibility of community pharmacies and their expanded role in public health services (Khayyat et al., 2024).

Despite its potential, the implementation of Raqeeb has not been without challenges. Pharmacists have reported administrative overload, lack of integration with existing pharmacy software, inadequate training, and concerns about regulatory scrutiny (Aldossari et al., 2025; Yasmeen et al., 2023). These operational difficulties are consistent with international experiences involving digital surveillance systems, where usability issues and system alert fatigue often undermine long-term adoption (Karavolis et al., 2022; Sheikh et al., 2022; Alzahrani et al., 2024). In rural or under-resourced areas, poor internet connectivity and limited technical infrastructure further restrict optimal use (Pomerleau et al., 2017).

Moreover, many pharmacists express concern about the limited availability of low-dose formulations, the absence of automated decision support tools, delays in insurance-covered prescriptions, and inconsistent feedback from regulators when concerns are flagged through the system (Goundrey-Smith, 2018; Yasmeen et al., 2023). Such challenges may reduce confidence in using Raqeeb, contribute to delays in patient care, or even discourage appropriate dispensing due to fear of regulatory repercussions.

To ensure the continued success of Raqeeb and similar national initiatives, it is crucial to understand the real-world perceptions and experiences of community pharmacists. This study aims to examine pharmacists’ knowledge, attitudes, and challenges in engaging with the Raqeeb platform. Findings are expected to guide system refinements, training needs, and policy adaptations that support safe and efficient NDPS monitoring in Saudi Arabia.

## 2 Methods

### 2.1 Study design and objectives

This study employed a quantitative, cross-sectional descriptive survey design using a structured, self-administered questionnaire to evaluate community pharmacists’ perceptions, practices, and challenges related to the use of the Raqeeb platform in Saudi Arabia. The primary objectives were to (Evoy et al., 2021) assess pharmacists’ awareness and attitudes toward the platform (Hägg et al., 2020), document the frequency and nature of their engagement with its features, and (Chiappini and Schifano, 2016) identify operational and regulatory barriers encountered during its use. The study was conducted between March and May 2025.

### 2.2 Study setting and participants

The study targeted licensed community pharmacists actively practicing in retail pharmacy settings (independent and chain pharmacies) across multiple regions of Saudi Arabia. Pharmacists working in hospital, military, or institutional pharmacies were excluded to ensure that participants had direct experience with the outpatient controlled substance dispensing process monitored by Raqeeb. Participants were recruited using convenience sampling through professional pharmacy networks, social media groups (e.g., WhatsApp, Telegram), and community pharmacy chains.

A total of 270 community pharmacists completed the survey. This number represents a significant proportion of the 747 registered community pharmacies in the Aseer region of Saudi Arabia, as reported by Alshahrani et al. (Alshahrani et al., 2021). Using the Raosoft sample size calculator with a 95% confidence level, 5% margin of error, and 50% response distribution, the minimum required sample size was calculated to be 254 pharmacists. Therefore, the achieved sample size of 270 ensures sufficient statistical power and enhances the generalizability of the findings within the regional context.

### 2.3 Survey instrument development

The questionnaire was developed in English following a comprehensive review of the literature on controlled substance regulation, digital prescription platforms, and community pharmacy practice. Prior evaluations of the Wasfaty system, international experiences with Prescription Drug Monitoring Programs (PDMPs), expert consensus on gabapentinoid regulation in Saudi Arabia, and studies assessing community pharmacists’ roles in medication safety and policy adherence informed its design (Alkhalaf et al., 2021; Althunian et al., 2022; Aldossari et al., 2025; Yasmeen et al., 2023; Alzahrani et al., 2024).

The final instrument consisted of five thematic sections (Evoy et al., 2021): demographic characteristics (Hägg et al., 2020), perceptions of the Raqeeb platform (Chiappini and Schifano, 2016), operational challenges (Pharmacopoeia, 2013), self-reported practice behaviors, and (Kurdi, 2021) attitudes toward potential improvements. A complete version of the questionnaire is now provided as Supplementary File S1 to enhance transparency and replicability.

To ensure face and content validity, the draft survey was reviewed by five experts in the fields of pharmacy practice, digital health, and healthcare regulation. A pilot test was conducted with 15 licensed community pharmacists to evaluate item clarity, completion time, and overall flow. Feedback from the pilot was used to refine item wording and layout. The final version of the instrument demonstrated acceptable internal consistency, with Cronbach’s alpha values exceeding 0.70 for the majority of the Likert-scale domains.

### 2.4 Data collection procedure

The survey was administered electronically via Google Forms. A cover page explained the study’s purpose, ensured participant anonymity, and included an informed consent statement. Only pharmacists who agreed digitally to participate and confirmed their current employment in a community pharmacy setting were able to proceed. No personally identifying information was collected, and duplicate responses were automatically restricted by the Google Forms settings.

### 2.5 Data analysis

Data collected from the online survey were downloaded and analyzed using IBM SPSS Statistics version 26. Descriptive statistics were employed to summarize participants’ demographic characteristics and responses to survey items. Categorical variables (e.g., gender, experience level, pharmacy type) were presented as frequencies and percentages.

Responses to Likert-scale items were analyzed as both means ± standard deviation and medians with interquartile ranges (IQR) to account for the ordinal nature of the data. Means are reported for comparability with prior studies, while medians/IQRs provide robustness.

The study was designed as descriptive, and no inferential statistical testing was prespecified or performed. Internal consistency and reliability of the Likert-scale domains (perception, challenges, practice) were assessed with Cronbach’s alpha coefficients, with values ≥ 0.70 considered acceptable.

### 2.6 Ethical considerations

The study protocol was reviewed and approved by the Research Ethics Committee at King Khalid University (Approval no. KKU-49-2025-17). Participation was voluntary, and all participants provided informed consent digitally before beginning the questionnaire. No personally identifiable information was collected, anonymity was guaranteed, and duplicate entries were prevented through form restrictions. Data were stored securely and analyzed in aggregate in accordance with the Declaration of Helsinki.

## 3 Results

### 3.1 Demographic characteristics of participants

A total of 270 community pharmacists completed the survey. The majority were male (58.1%), aged between 20 and 39 years (74.8%), and 62.2% held a PharmD degree. Most respondents (78.1%) had less than 5 years of experience, 89% worked in chain pharmacies, and 70% practiced in urban areas. This demographic profile indicates an overrepresentation of younger and urban pharmacists, which is consistent with the sampling approach and acknowledged as a study limitation. Demographic details are presented in Table 1.

**TABLE 1:** Demographic characteristics of participants.

Demographic Variable	Category	Frequency	Percentage (%)
Gender	Male	157	58.1
	Female	113	41.9
Age group	20–29	110	40.7
	30–39	92	34.1
	40–49	32	11.9
	50+	8	3
Education	Bachelor	70	25.9
	PharmD	168	62.2
	Postgraduate	32	11.9
Experience	<5 years	211	78.1
	5–10 years	35	13
	>10 years	24	8.9
Pharmacy type	Independent	30	11.1
	Chain	240	88.9
Location	Urban	189	70
	Rural	81	30
Used Raqeeb in last 6 Months	Yes	103	38.1
	No	167	

### 3.2 Combined factors affecting pharmacists’ experience with raqeeb

Survey responses on pharmacists’ knowledge, attitudes, and challenges are summarized in Table 2. The majority of respondents reported high awareness of the platform’s purpose and its contribution to controlled medication safety. Confidence in navigating the system was moderate, while overall attitudes were favorable, with strong support for mandatory training, nationwide expansion, and integration with existing pharmacy systems.

**TABLE 2:** Pharmacists’ knowledge, attitudes, and challenges responses related to the raqeeb platform.

Domain	Item	Strongly Agree (5)	Agree (4)	Neutral (3)	Disagree (2)	Strongly Disagree (1)	Skew	Mean	SD
Knowledge	I am aware of the purpose and goals of the Raqeeb platform	73 (27.0%)	115 (42.6%)	45 (16.7%)	25 (9.3%)	12 (4.4%)	−0.86	3.79	1.08
	I understand how Raqeeb contributes to controlled medication safety	65 (24.1%)	125 (46.3%)	50 (18.5%)	20 (7.4%)	10 (3.7%)	−0.89	3.8	1.01
	I feel confident navigating the Raqeeb interface	60 (22.2%)	100 (37.0%)	60 (22.2%)	32 (11.9%)	18 (6.7%)	−0.6	3.56	1.15
Attitude	Raqeeb should be integrated with pharmacy systems	67 (24.8%)	115 (42.6%)	55 (20.4%)	22 (8.1%)	11 (4.1%)	−0.8	3.76	1.04
	Mandatory Raqeeb training should be provided to pharmacists	89 (33.0%)	120 (44.4%)	40 (14.8%)	15 (5.6%)	6 (2.2%)	−1.02	4	0.95
	Raqeeb contributes positively to public health policy	62 (23.0%)	110 (40.7%)	56 (20.7%)	28 (10.4%)	14 (5.2%)	−0.71	3.66	1.1
	Raqeeb improves accountability in controlled drug dispensing	65 (24.1%)	112 (41.5%)	54 (20.0%)	26 (9.6%)	13 (4.8%)	−0.76	3.7	1.08
	I support the nationwide expansion of Raqeeb functionalities	74 (27.4%)	120 (44.4%)	47 (17.4%)	20 (7.4%)	9 (3.3%)	−0.9	3.85	1.01
Challenge	The Raqeeb system interface is difficult to navigate	45 (16.7%)	65 (24.1%)	60 (22.2%)	58 (21.5%)	42 (15.6%)	−0.06	3.05	1.32
	I have not received adequate training to use Raqeeb	42 (15.6%)	50 (18.5%)	70 (25.9%)	60 (22.2%)	48 (17.8%)	0.09	2.92	1.32
	I fear legal consequences from using Raqeeb incorrectly	53 (19.6%)	60 (22.2%)	72 (26.7%)	49 (18.1%)	36 (13.3%)	−0.14	3.17	1.3
	Raqeeb causes delays in daily pharmacy workflow	50 (18.5%)	58 (21.5%)	67 (24.8%)	55 (20.4%)	40 (14.8%)	−0.06	3.09	1.32
	There is insufficient technical support for Raqeeb users	55 (20.4%)	70 (25.9%)	65 (24.1%)	45 (16.7%)	35 (13.0%)	−0.25	3.24	1.31
	Raqeeb lacks integration with my pharmacy’s software system	53 (19.6%)	66 (24.4%)	63 (23.3%)	50 (18.5%)	38 (14.1%)	−0.17	3.17	1.32
	Some controlled drugs are unavailable through Raqeeb	63 (23.3%)	75 (27.8%)	60 (22.2%)	42 (15.6%)	30 (11.1%)	−0.37	3.37	1.3

Despite these positive perceptions, key challenges were noted: difficulties with the interface, inadequate training, lack of software integration, and the unavailability of certain controlled medications. These barriers were endorsed by a substantial proportion of participants, indicating practical issues that may hinder optimal implementation (see Table 2).

### 3.3 Internal consistency and distribution by domain

Domain-level statistics are presented in Table 3, including means, medians, interquartile ranges (IQR), skewness, and Cronbach’s alpha coefficients. Knowledge and Attitude domains scored higher, reflecting favorable perceptions and strong support for Raqeeb, with Cronbach’s alpha values (0.81 and 0.87) confirming good reliability.

**TABLE 3:** Distribution and internal consistency by domain.

Description of Scale	≤1	≤2	≤3	≤4	≤5	Skew	Mean	Median (IQR)	SD	Cronbach α
Knowledge	4.93	14.43	33.56	80.9	100	−0.78	3.72	3.80 (3.0–4.0)	1.08	0.81
Attitude	3.92	12.14	30.8	73.54	100	−0.84	3.81	3.90 (3.0–4.0)	1.03	0.87
Challenge	14.24	33.23	57.4	75.56	100	−0.18	3.16	3.00 (2.0–4.0)	1.31	0.73

In contrast, the Challenge domain had lower mean and median scores with moderate reliability (α = 0.73), suggesting that although pharmacists consistently identified barriers, responses were more variable. This indicates specific areas for system improvement that require targeted interventions. Table 3 summarizes the distribution and internal consistency by domain.

## 4 Discussion

The Raqeeb platform is a transformative leap forward in Saudi Arabia’s public health strategy to combat the misuse of narcotic drugs and psychotropic substances (NDPS), particularly gabapentinoids like pregabalin and gabapentin, which have seen rising recreational use globally and locally (Aldossari et al., 2025). As a cornerstone of Saudi Arabia’s Vision 2030, Raqeeb leverages digital innovation to enhance healthcare transparency, safety, and regulatory compliance through real-time monitoring of NDPS prescribing and dispensing (Aldossari et al., 2025; Al-Kahtani et al., 2022). This study provides new insights into community pharmacists’ engagement with Raqeeb, highlighting both strengths and practical challenges. Importantly, the findings position Raqeeb not only as a regional model but also as a system with global relevance if usability, integration, and training barriers are addressed.

Pharmacists’ robust awareness of Raqeeb’s purpose and its contribution to medication safety mirrors the success of Australia’s SafeScript program, which achieved a 20% reduction in opioid-related overdoses by enhancing prescriber accountability through real-time data monitoring (Alzahrani et al., 2024). Similarly, the UK’s Electronic Prescription Service (EPS) has improved dispensing accuracy by integrating digital prescribing tools across healthcare sectors, a goal Raqeeb aims to replicate (Goundrey-Smith, 2018; Sheikh et al., 2022). However, unlike international systems, Saudi-specific policy considerations must be emphasized. In particular, integration with Seha (the national digital health platform) and with insurance systems is essential to achieve seamless adoption and maximize utility for pharmacists and patients.

Challenges such as a complex interface and a lack of integration with existing pharmacy software align with global PDMP experiences. In the U.S., studies report that 35%–40% of pharmacists find PDMP interfaces cumbersome, leading to reduced adoption rates and workflow inefficiencies (Pomerleau et al., 2017; Haffajee et al., 2018; Alshehri et al., 2023; Lin et al., 2017). Raqeeb’s integration issues, noted by 44% of respondents, highlight the need for seamless interoperability. User-centered design principles should guide future upgrades to ensure the platform complements pharmacists’ workflows. In addition, previous studies have shown that electronic prescribing services (EPS) reduce medication errors and improve healthcare delivery, with 74% of patients preferring EPS over paper-based prescriptions (Shams, 2011). The integration of EPS with hospital information systems has led to reduced prescribing errors, as reported by physicians, pharmacy staff, and nurses (Shams, 2011). However, challenges persist, including missing electronic prescriptions and design-specific issues (Mozaffar et al., 2017). These parallels reinforce that usability must be a central design priority for Raqeeb, supported by a strong technical infrastructure.

The strong support for mandatory Raqeeb training (77.4%) and nationwide expansion (71.8%) reflects pharmacists’ recognition of its public health value, consistent with studies on Saudi Arabia’s Wasfaty system, where structured training boosted adoption (Yasmeen et al., 2023; Alzahrani et al., 2024). Internationally, pharmacists with comprehensive PDMP training were significantly more likely to use monitoring systems effectively (Lin et al., 2017). Raqeeb’s training gaps, with 40.7% citing inadequate preparation, underscore the need for robust, mandatory training programs. Adopting structured and continuous training initiatives—potentially linked with professional licensure or CPD requirements would enhance pharmacists’ ability to engage with Raqeeb confidently. Canada’s PharmaNet system, where training supported reduced inappropriate prescribing of opioids and benzodiazepines, provides a strong precedent (Pontefract et al., 2018; Fleming et al., 2014; Dormuth et al., 2012).

Operational challenges, including medication unavailability (51.1%) and workflow delays, echo issues observed in France’s gabapentinoid monitoring program, where supply chain gaps reduced dispensing efficiency (Driot et al., 2019). In Saudi Arabia, the specific causes of unavailability warrant further investigation to determine whether they are related to supply chain disruptions, regulatory restrictions, or technical limitations within Raqeeb. Addressing this gap is critical to ensure that controlled medications are accessible to patients without compromising regulatory oversight. Connectivity and infrastructure barriers, particularly in rural areas, may further compound adoption difficulties, consistent with national reports of e-health implementation challenges (Almuayqil et al., 2016; Martin et al., 2021). Fear of regulatory scrutiny, noted by 41.8% of Raqeeb users, parallels U.S. findings where pharmacists hesitated to dispense controlled substances due to legal concerns (Radomski et al., 2018; Cochran et al., 2016; Peckham et al., 2018; Coustasse et al., 2025). Australia’s Real Time Prescription Monitoring program addressed this by providing clear regulatory guidelines, reducing apprehension, and improving compliance (Dobbin and Liew, 2020). For Saudi Arabia, transparent legal frameworks and supportive communication from regulators could similarly reduce pharmacists’ concerns and promote confidence in dispensing practices.

To elevate Raqeeb’s efficacy, policymakers should prioritize user-centered design, integration with existing software, technical support, and medication availability. Visual decision-support features and dashboard summaries could further streamline workflows and facilitate rapid interpretation of controlled substance use patterns. Recent studies highlight the evolving role of pharmacists in global healthcare systems, emphasizing the need for advanced training and integration into primary care teams (Anderson et al., 2009; Wagner et al., 2023).

### 4.1 Limitations

This study has several limitations. First, the use of convenience sampling through professional networks and social media may have introduced selection bias, leading to overrepresentation of younger and urban pharmacists. This limits the generalizability of the findings to all community pharmacists in Saudi Arabia. Second, the study was designed as a descriptive survey and did not prespecify inferential analyses; thus, subgroup comparisons (e.g., by gender, years of experience, or practice setting) could not be conducted. Third, although the survey instrument underwent validation and demonstrated good internal consistency, self-reported data are subject to recall and social desirability bias. Finally, the cross-sectional design captures pharmacists’ perceptions at a single point in time and does not allow causal inference.

Despite these limitations, the study provides valuable first evidence on pharmacists’ engagement with Raqeeb and offers actionable recommendations for policy and practice. By incorporating these lessons, Raqeeb has the potential to evolve from an early-stage innovation into a globally recognized model for controlled substance monitoring that considers both Saudi-specific policy priorities and international best practices.

## 5 Conclusion

The Raqeeb platform represents a pivotal advancement in Saudi Arabia’s strategy to combat the misuse of narcotic drugs and psychotropic substances, aligning with Vision 2030s digital healthcare transformation goals. This study demonstrated that community pharmacists generally support Raqeeb’s role in enhancing medication safety and accountability, while also identifying practical barriers that may hinder its optimal adoption. Key challenges include system usability, insufficient training, and medication unavailability. Addressing these will require user-centered redesign, integration with existing pharmacy software, and comprehensive training programs, ideally linked with continuous professional development. In addition, ensuring reliable medication supply and providing clear regulatory guidance will be essential for strengthening pharmacists’ confidence in dispensing controlled substances. Even though international systems such as SafeScript and EPS provide useful lessons, the future success of Raqeeb depends on tailoring solutions to Saudi-specific contexts, including integration with Seha, collaboration with insurers, and engagement with local stakeholders. By implementing these targeted improvements, Raqeeb can progress from its current early stage to a global exemplar of digital prescription monitoring, reinforcing Saudi Arabia’s leadership in innovative, technology-enabled healthcare solutions.

## Data Availability

The raw data supporting the conclusions of this article will be made available by the authors, without undue reservation.
