# Supplementary material for: Community pharmacists’ engagement with Raqeeb platform: perceptions and implementation challenges toward controlled substance monitoring in Saudi Arabia
Source: Front Pharmacol. 2025 Sep 25;16:1644493. doi: 10.3389/fphar.2025.1644493 (PMC12507632; doi:10.3389/fphar.2025.1644493)
Supplement: Supplementary file 1 [file Supplementaryfile1.pdf]

Community Pharmacists' Engagement with Raqeeb Platform: Perceptions and Implementation Challenges Toward Controlled Substance Monitoring in Saudi Arabia

We are a group of researchers in the clinical pharmacy department at King Khalid University conducting a research project entitled "Community Pharmacists' Engagement with Raqeeb Platform: Perceptions and Implementation Challenges Toward Controlled Substance Monitoring in Saudi Arabia". The purpose of this project is to investigate Community Pharmacists' Engagement with Raqeeb Platform: Perceptions and Implementation Challenges Toward Controlled Substance Monitoring in Saudi Arabia. Your participation in this research project is voluntary. You may choose not to participate. If you decide to participate in this research survey, you may withdraw at any time. The procedure involves filling a survey that will take approximately 4-5 minutes. Your responses will be confidential, and we do not collect identifying information such as your name, email address or IP address.

Consent:

Before starting to answer the survey, I am fully aware that my participation is fully voluntary, and I can withdraw from the survey without any penalties

A. Agree, continue the survey. **B. Disagree, and withdraw from the survey**

Survey

Section 1: Demographic Information

1. **Gender**
   ☐ Male ☐ Female
2. **Age Group**
   ☐ 20–29 ☐ 30–39 ☐ 40–49 ☐ 50+
3. **Educational Qualification**
   ☐ Bachelor's Degree ☐ PharmD ☐ Postgraduate Degree
4. **Years of Experience**
   ☐ <5 years ☐ 5–10 years ☐ >10 years
5. **Pharmacy Type**
   ☐ Independent ☐ Chain
6. **Location of Pharmacy**
   ☐ Urban ☐ Rural
7. **Region**
   ☐ Aseer ☐ Other (please specify): ___________

8. **Have you used the Raqeeb platform in the past 6 months?**
☐ Yes ☐ No

Section 2: Knowledge About the Raqeeb Platform

(Please indicate your level of agreement with each statement)

Statement	Strongly Disagree	Disagree	Neutral	Agree	Strongly Agree
9. I am aware of the purpose and goals of the Raqeeb platform.	☐	☐	☐	☐	☐
10. I understand how Raqeeb contributes to controlled medication safety.	☐	☐	☐	☐	☐
11. I feel confident navigating the Raqeeb interface.	☐	☐	☐	☐	☐

Section 3: Attitudes Toward the Raqeeb Platform

Statement	Strongly Disagree	Disagree	Neutral	Agree	Strongly Agree
12. Raqeeb should be integrated with pharmacy systems.	☐	☐	☐	☐	☐
13. Mandatory Raqeeb training should be provided to pharmacists.	☐	☐	☐	☐	☐
14. Raqeeb contributes positively to public health policy.	☐	☐	☐	☐	☐
15. Raqeeb improves accountability in controlled drug dispensing.	☐	☐	☐	☐	☐
16. I support the nationwide expansion of Raqeeb functionalities.	☐	☐	☐	☐	☐

Section 4: Challenges in Using the Raqeeb Platform

Statement	Strongly Disagree	Disagree	Neutral	Agree	Strongly Agree
17. The Raqeeb system interface is difficult to navigate.	☐	☐	☐	☐	☐
18. I have not received adequate training to use Raqeeb.	☐	☐	☐	☐	☐
19. I fear legal consequences from using Raqeeb incorrectly.	☐	☐	☐	☐	☐
20. Raqeeb causes delays in daily pharmacy workflow.	☐	☐	☐	☐	☐
21. There is insufficient technical support for Raqeeb users.	☐	☐	☐	☐	☐

Statement	Strongly Disagree	Disagree	Neutral	Agree	Strongly Agree
22. Raqeeb lacks integration with my pharmacy’s software system.	☐	☐	☐	☐	☐
23. Some controlled drugs are unavailable through Raqeeb.	☐	☐	☐	☐	☐

Section 6: Suggestions for Improvement

1. What improvements or support would help you use Raqeeb more effectively? (Open-ended)
